# Heavy metals and organic compounds contamination in leachates collected from Deir Kanoun Ras El Ain dump and its adjacent canal in South Lebanon

**DOI:** 10.1016/j.heliyon.2019.e02212

**Published:** 2019-08-21

**Authors:** Jamilah Borjac, Manal El Joumaa, Rawan Kawach, Lobna Youssef, Diane A. Blake

**Affiliations:** a Department of Biological Sciences, Beirut Arab University, Debbieh, Lebanon; b Department of Chemistry, Beirut Arab University, Debbieh, Lebanon; c Department of Biochemistry and Molecular Biology, Tulane University School of Medicine, New Orleans, LA, USA

**Keywords:** Environmental science, Dump, Contamination, Heavy metals, Organic compounds, Water pollution

## Abstract

Environmental pollution generated from uncontrolled dumping is a major problem in Lebanon due to the lack of proper waste management plans. Deir Kanoun Ras El Ain is the village that harbors the worst dumps in Lebanon. Wastewater leachates of this dump influx into an adjacent nearby canal used for irrigation and drinking purposes. The aim of this study is to assess the concentrations of heavy metals (Pb, Cd, As, and Hg) and the presence of organic compounds (phthalates, bisphenol A, and polycyclic aromatic hydrocarbons (PAHs)) in water samples collected from two different sites around the dump and two canal sites during winter and summer seasons. The concentrations of heavy metals were determined using atomic absorption spectrophotometry, while the identification of the extracted organic compounds was performed using High Performance Liquid Chromatography coupled to Mass Spectrometry (HPLC–MS). The carried analyses revealed that water samples collected from dump and canal were heavily polluted by Cd, As, Hg, phthalates, bisphenol A, and PAHs caused by pyrogenic and petrogenic sources. The concentrations of the found heavy metals were far above the maximum tolerable levels set by different guidelines. The findings suggest that the studied water sources are not safe for irrigation and drinking. The serious implications of dumping wastes on the health of inhabitants recall for an immediate employment of efficient waste management policies to resolve this problem.

## 1 Introduction

Human activities, as translated in the form of environmental pollution, are causing major geochemical transformations to nature [1]. Unregulated dumping and burning of wastes in many countries cause serious contamination of the surrounding environment and lead to intensive soil, water and atmosphere pollution. In Lebanon, the failure to develop and carry out a long-term national waste management plan based on public health principles has led to hundreds of open dumps, and the problem is exacerbated with open burning at many of these waste sites [2, 3, 4, 5]. Contamination from these dumps includes heavy metals (Pb, Cd, As, and Hg) as well as organic compounds such as phthalates, bisphenols and polycyclic aromatic hydrocarbons (PAHs) [6, 7]. Inhabitants who live near the dumps are exposed to these toxic compounds through dermal contact or ingestion of contaminated water. Once in the body, these contaminants accumulate in various tissues and may reach critical levels that give rise to acute intoxications and health problems [8, 9].

**Fig. 1:**
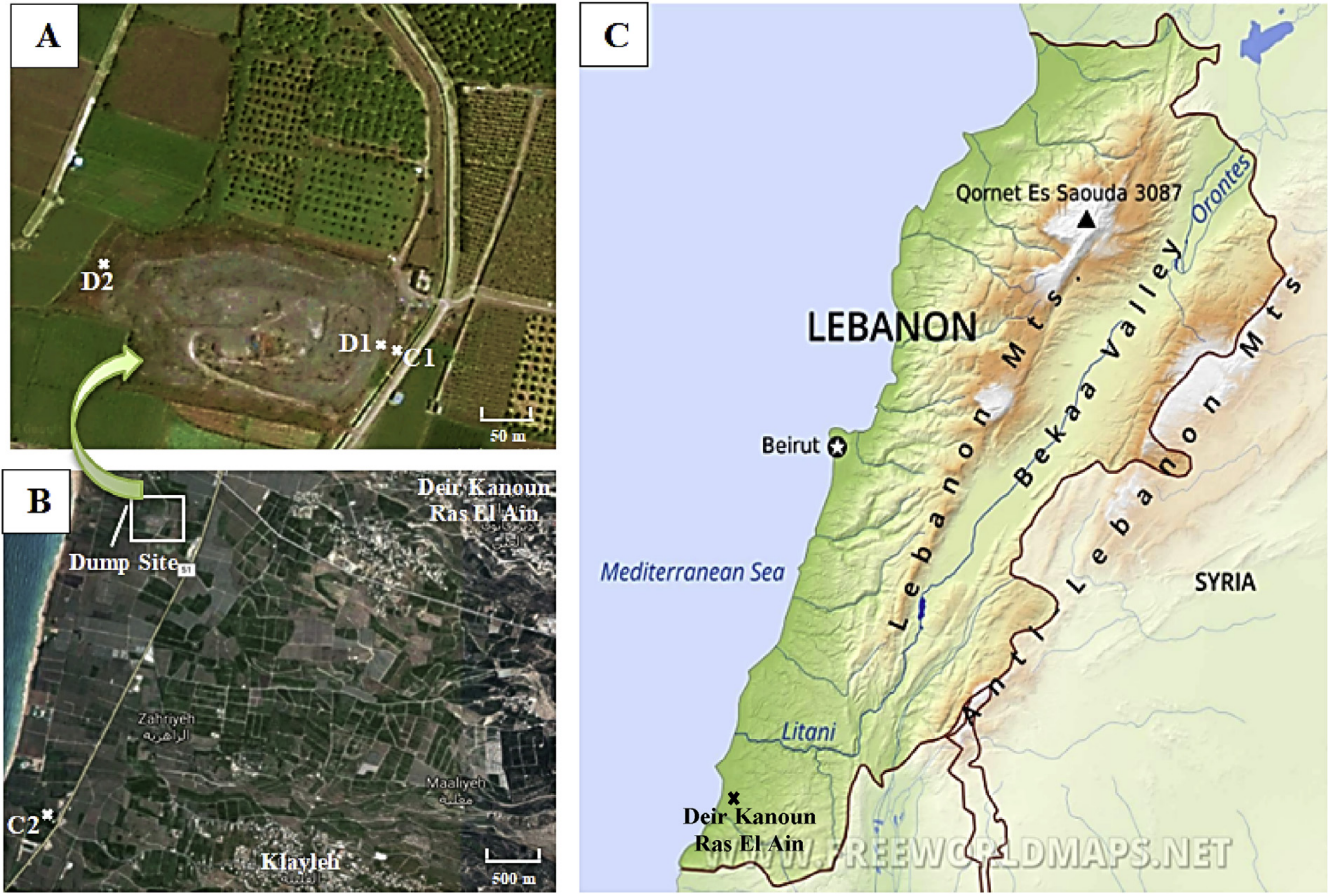
A & B: Pictures of the study area showing the dump and canal sites (D1, D2, C1, and C2) from where samples were collected; C: Map of Lebanon showing Deir Kanoun Ras El Ain village.

Deir Kanoun Ras El Ain is a southern Lebanese village that possesses significant underground water resources used to both irrigate agricultural lands and provide the village's residents with drinking water. Unfortunately, it also has one of the worst dumps in the country, where medical, industrial and household wastes have been deposited. Wastes from many surrounding villages and as well as from the Palestinian refugees camps were thrown in this dump for over than 20 years. The dump has an area of 12230m^2^, and an estimated volume of 183,450 m^3^. The dump was closed at the end of the year 2015. Fig. 1 shows the location of the dump in the middle of an agricultural area. No physical space separates the effluxing leachate from neither the surrounding agricultural soil nor the nearby canal that irrigate these lands. This dump poses detrimental health impacts on both local inhabitants and Syrian Refugees living in camps in this region [10]. In this study, water samples were collected from this dump and from the canal running along it during two seasons. Samples were tested for heavy metals and organic compounds.

## 2 Materials and methods

### 2.1 Site and sample collection

The sampling activities from the dump and canal were conducted during the winter and summer seasons of the year 2017 (Fig. 1). Four sampling sites were selected. Two sites were from the canal where the dump's leachate is influxing. The first canal sampling site (C1) was in direct contact with dump (a short stone barrier separating the dump from the canal) and the second sampling site (C2) is around 3 km away. Dump leachates were collected in two sampling sites, i.e. D1, located ca. 2.5 m away from C1, and D2, on the opposite side of the dump with respect to D1 (Table 1; Fig. 1). Liquid samples (in triplicate) were taken from surface of canal and leachates during both seasons while samples from dump were only collected during the winter season due to complete dryness in the summer season. All samples were collected during the same day. Autoclaved bottles pre-washed with 10% (v/v) nitric acid and rinsed in deionized water were used for liquid sample collection. Samples used to assess heavy metal content were acidified with concentrated nitric acid (0.5:250 v/v) soon after collection while those used to assess organic compounds were not. The collection was performed according to the WHO standard methods/APHA [11]. Samples were stored at 4 °C prior to analysis.

**Table 1:** Sampling sites from Deir Kanoun dump and canal.

Site	Location details
C1	• Deir Kanoun Canal • **Latitude (N):** 33^o^ 13′ 43.77″; **Longitude (E):** 35^o^ 15′ 48.061″
C2	• Klayleh • **Latitude (N):** 33^o^ 28′ 48.989″; **Longitude (E):** 35^o^ 20′ 20.399″
D1	• Dump Contact with C1 • **Latitude (N):** 33^o^ 13′ 43.77″; **Longitude (E):** 35^o^ 15′ 48.061″
D2	• Dump around 220^o^ to C1 • **Latitude (N):** 33^o^ 13′ 43.453″; **Longitude (E):** 35^o^ 13′ 33.628″

### 2.2 Heavy metals analysis

Concentration of heavy metals (Pb, Cd, As, and Hg) was determined using atomic absorption spectrophotometer (Thermo Fisher Scientific, Model: GF95Z, UK) at the Lebanese Agricultural Research Institute (LARI) in Beirut, Lebanon.

### 2.3 Organic compounds analysis

All samples were filtered using a 0.45 μm membrane filters (Whatmann) before extraction of the specified organic compounds.

#### 2.3.1 Phthalates extraction

Phthalates were extracted according to Hadjmohammadi *et al.* [12]. In brief, water samples (5 mL) were mixed with the extracting solvent (methanol/chloroform, 1:4 v/v) at a ratio of sample to solvent of 5:2 (v/v). The mixture was salted out with 1g of NaCl and then centrifuged for 3 min at 5000 rpm. Sediments were evaporated to dryness. Residues were then dissolved in methanol. Phthalates were separated by High Performance Liquid Chromatography (HPLC) using an ODS-C18 (25 cm × 4.6 mm, 5 μm) column where the mobile phase consisted of acetonitrile and water at a ratio of 3:1 (v/v) at a flow rate 1 mL/min. Absorbance of the eluted products was measured at 226 nm [13].

#### 2.3.2 Bisphenol A extraction

Extraction of bisphenol A from water samples was according to Rezaee *et al* [14]. Water samples were mixed with a solvent mix consisting of acetone and chloroform at a ratio of 2:3 (v/v). Samples were then centrifuged for 5 min at 6000 rpm. Dried sediments obtained after solvent evaporation were re-dissolved in HPLC-grade methanol before separation on a Nucleosil-C18 (25 cm × 4.6 mm, 5 μm) column using acetonitrile and water (50:50, v/v) as a mobile phase. Flow rate was at 1 mL/min and eluted products were monitored by absorbance 275 nm [15].

#### 2.3.3 Polycyclic aromatic hydrocarbons (PAHs) extraction

PAHs were extracted by sonication for 30 min with dichloromethane (DCM) followed by rotary-evaporation. Pellets were re-dissolved in hexane prior to separation by HPLC on a C18 (25 cm × 4.6 mm, 5 μm) column using acetonitrile and water (70:30, v/v) as a mobile phase at a flow rate of 0.8 mL/min. Absorbance of eluted products was monitored at 220 nm [17].

### 2.4 Statistical analysis

For data analysis, SPSS software (SPSS for Windows, Version 23.0, SPSS Inc., Chicago, IL, USA) was used. Correlation matrix between the different heavy metals was assessed using bivariate correlation test and Pearson correlation coefficient.

## 3 Results

### 3.1 Heavy metals

**Fig. 2:**
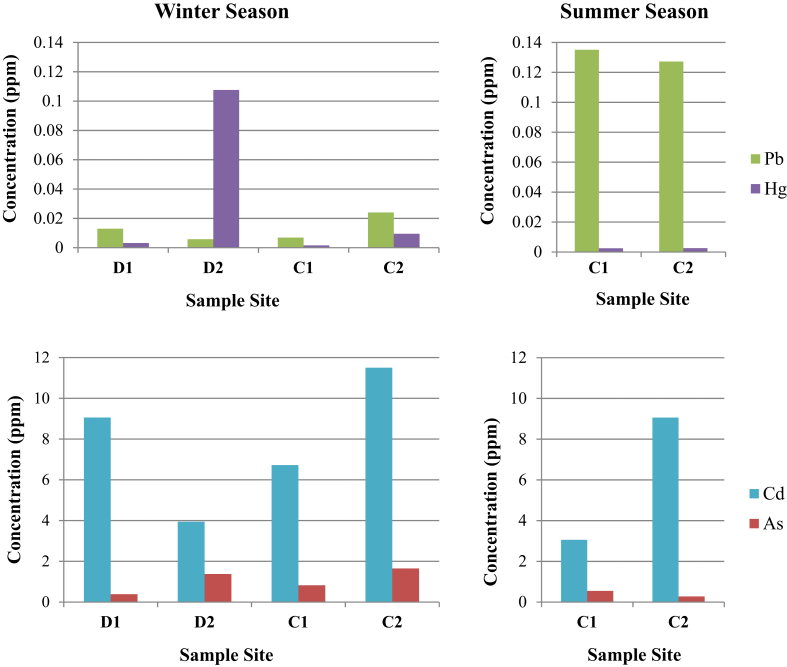
Variation of heavy metals concentrations in water samples from different sites during winter and summer seasons.

The average heavy-metal concentrations (ppm) in water samples collected during winter (W) and summer (S) seasons are summarized in Table 2. Fig. 2 also shows the variation of heavy metals in samples collected from different sites during the two seasons.

**Table 2:** Average concentrations of heavy metals in water collected from Deir Kanoun dump and canal.

Heavy Metal (ppm)	Sample Site							FAO Standards ppm^a^	CCME Standards ppm^b^	WHO Drinking Water Standards
	D1	D2	C1			C2				
	W	W	W		S	W	S			
Pb	0.0129 ± 0.0012	0.0057 ± 0.0008	0.0068 ± 0.0012	0.135 ± 0.003		0.024 ± 0.008	0.127 ± 0.002	5	0.2	0.01
Cd	9.056 ± 1.763	3.944 ± 0.667	6.722 ± 0.314	3.056 ± 0.660		11.5 ± 0.778	9.056 ± 0.044	0.01	0.0051	0.003
As	0.384 ± 0.138	1.373 ± 0.189	0.824 ± 0.078	0.549 ± 0.107		1.648 ± 0.599	0.274 ± 0.159	0.1	0.1	0.01
Hg	0. 0032 ± 0.0006	0.1076 ± 0.0098	0.0016 ± 0.0012	0.0249 ± 0.0011		0.0095 ± 0.0035	0.0253 ± 0.0032	0.001	No data	0.001

a Maximum levels allowed for agricultural irrigation; Food and Agricultural Organization of the United Nations [18].

b Maximum levels allowed for agricultural irrigation; Canadian Council of Ministers of the Environment [19].

During winter season, Pb, Cd, As, and Hg were abundant in the water samples collected from dump and canal, where their concentrations ranged between 0.0057-0.024 ppm, 3.056–11.5 ppm, 0.384–1.648 ppm, and 0.0016–0.1076 ppm respectively. On average, the highest Pb content (0.024 ppm) was found in water samples from C2 site compared to other samples. The concentrations of Pb, As, Cd and Hg at C2, which is the furthest site from the dump, showed an increasing pattern when compared to those at C1. As for the samples collected from the dump sites, D1 had higher concentrations of Pb and Cd (0.0129 and 9.056 ppm respectively) than D2. On the other hand, D2 had higher concentrations of As and Hg (1.373 and 0.1076 ppm respectively) than D1.

The concentrations of Cd, Hg, and As, which are among the most serious metal pollutants in the water samples collected from Deir Kanoun dump and canal, exceeded the FAO [18] and CCME standard values [19], while only the levels of Pb were below these standards.

During summer season, the highest concentrations of Pb and As were found in water samples from C1 site (0.135 and 0.549 ppm respectively), whereas the highest Cd concentration (9.056 ppm) was observed in C2 samples. Hg concentration in C1 samples was comparable to that from C2 site. Similar to winter collections, all sites showed high levels of metal contamination that greatly exceed the FAO and CCME standards, except for Pb.

### 3.2 Correlation matrix

The variations of heavy metals in water samples were evaluated during the winter season in a correlation matrix using the Pearson correlation coefficient. Table 3 shows the correlation matrix that assesses the variance of each heavy metal in relationship with each of the others.

**Table 3:** Correlation matrix between the different heavy metals.

Heavy Metal	Pb	Cd	As	Hg
Pb	1			
Cd	**0.928**	1		
As	**0.402**	0.068	1	
Hg	-0.472	-0.757	**0.425**	1

The lead/cadmium association (r = 0.928) indicates a strong positive correlation between these metals. Moreover, lead has a moderate correlation with arsenic (r = 0.402), and arsenic has a moderate correlation with mercury (r = 0.425). These associations may be due to the inputs of plastic and pesticide wastes that are common sources of these heavy metals [6].

### 3.3 Organic compounds

**Fig. 3:**
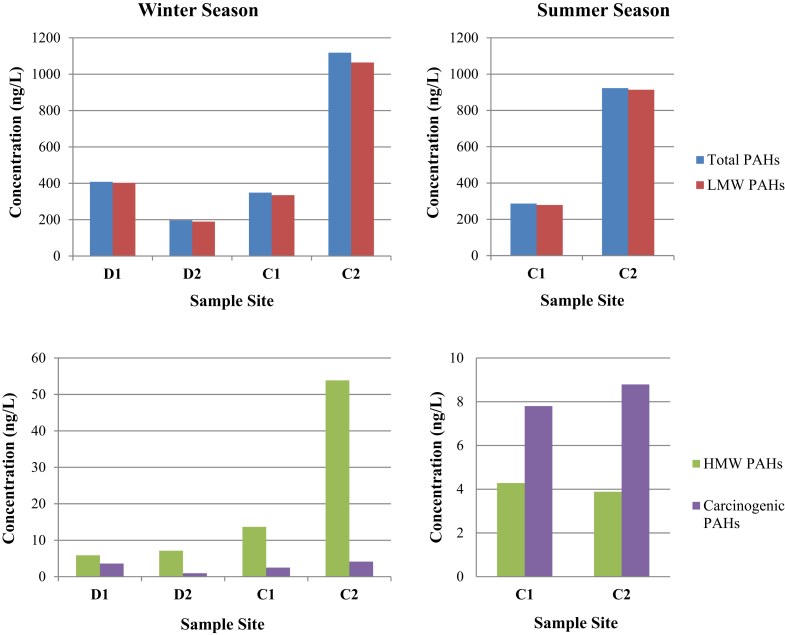
Variation of total, LMW, HMW, and carcinogenic PAHs concentrations in water samples from different sites during winter and summer seasons.

Determining the concentrations of organic compounds in water samples collected from the dump and canal provided insights toward identifying the source of contamination. As seen in Table 4, all water samples contained phthalates (DEHP), bisphenol A as well as PAHs including naphthalene, 2-methyl-naphtalene, 1-methyl-naphtalene, fluorene, phenanthrene, fluoranthene, pyrene, and chrysene. The variation of total, low molecular weight (LMW), high molecular weight (HMW), and carcinogenic PAHs among samples from different sites is illustrated in Fig. 3.

**Table 4:** Average concentrations of organic compounds in water samples collected from Deir Kanoun dump and canal during winter and summer seasons.

Organic compound	Sample site					
	D1	D2	C1		C2	
	W	W	W	S	W	S
Bis(2-ethylhexyl) phthalate DEHP (μg/L)	110.2	-	85.05	72.3	89.2	-
Bisphenol A (μg/L)	0.012	-	0.183	0.154	0.193	-
**PAHs (ng/L)**						
Napthalene	108.97	73.24	106.86	86.26	459.49	199.71
2-Methyl-naphthalene	163.94	52.52	57.15	96.34	342.61	248.21
1-methyl-naphthalene	80.45	24.68	44.79	66.18	192.42	433.23
Fluorene	11.8	5.94	18.89	5.33	58.96	3.69
Phenanthrene	25.42	32.77	107.19	24.64	10.73	29.05
Anthracene	11.59	-	-	-	-	-
Fluoranthene	0.99	1.39	3.62	2.12	2.48	3.04
Pyrene	1.27	1.38	3.27	1.40	2.67	1.87
Chrysene^a^	3.59	0.93	2.5	1.25	0.626	0.86
Acenaphthlene	-	-	-	-	29.67	-
Acenaphthylene	-	3.42	4.26	-	14.92	-
Benzo(a)anthracene^a^	-	-	-	1.65	0.229	1.36
Benzo(b)fluoranthene^a^	-	-	-	1.38	1.06	1.45
Benzo(k)fluoroanthene^a^	-	-	-	-	0.656	0.21
Benzo(a)pyrene^a^	-	-	-	-	0.831	-
Benzo(g,h,i)perylene^a^	-	-	-	-	0.71	-
Total PAHs	408.02	196.27	348.53	286.55	1118.06	922.68
Carcinogenic PAHs	3.59	0.93	2.5	4.28	4.11	3.88
LMW PAHs	402.17	189.15	334.88	278.75	1064.21	913.89
HMW PAHs	5.85	7.12	13.65	7.8	53.85	8.79
**Ratios**						
Phe/Ant	2.193	-	-	-	-	-
Flt/Pyr	0.779	1.007	1.107	1.514	0.928	1.625
Flt/Flt + Pyr	0.438	0.502	0.525	0.602	0.482	0.619
Ant/Ant + Flt	0.313	-	-	-	-	-

(-): concentration falls below the detection limit of the instrument.

a refers to carcinogenic PAHs.

During winter season, the levels DEHP and bisphenol A ranged from 83.3-110.2 μg/L and 0.012–0.193 μg/L. Highest level of DEHP were found in samples collected from D1 site, while highest bisphenol A level was found in samples from C2 site. On the other hand, samples from D4 site had undetectable levels of DEHP and bisphenol A.

In addition, total PAHs content ranged from 196.27 ng/L (at D2) to 1118.06 ng/L (at C2) with C2 site being highly contaminated with PAHs. Naphthalene showed the highest concentrations among other PAHs, followed by 2-methyl-naphtalene, 1-methyl-naphtalene, fluorene, phenanthrene, fluoranthene, pyrene, and chrysene. Other less abundant PAHs such as acenaphthlen, benzo(a)anthracene, benzo(b)fluoranthene, benzo(k)fluoroanthene, and benzo(a)pyrene, were particularly detected in water samples from C2 site. Benzo(g,h,i) perylene was detected in samples from C2 site only.

Low molecular weight PAHs, which originate mainly from petrogenic sources, are made up of less than four aromatic rings such as naphthalene, acenaphthene, acenaphthylene, fluorene, phenanthrene, and anthracene [20]. In the water samples under study herein, they accounted for 95.2–98.5% of the total PAHs concentration. On the other hand, high molecular weight PAHs, implying pyrolytic sources of PAHs, are made up of four or more aromatic rings and include flouranthene, pyrene, chrysene, dibenz anthracene, benz[a]anthracene, benzo[b]fluoranthene, benzo[k]fluoranthene, benzo[a]pyrene, and benzo[g,h,i]perylene [20, 21]. They accounted for 1.43%–4.81% of total PAHs. In general, there was higher abundancy of low molecular weight PAHs than high molecular weight PAHs in the samples tested. PAHs profile in the water leachates collected from dump and canal suggested the presence of both petrogenic and pyrolytic sources of PAHs.

Moreover, calculating PAH fingerprint ratios of phenanthrene/anthracene (Phe/Ant), fluoranthene(Flt)/pyrene(Pyr) (Flt/Pyr), anthracene/(antracene + phenanthrene) (Ant/(Ant + Phe)), and fluoranthene/(fluoranhtene + pyrene) (Flt/(Flt + Pyr)) were performed to further infer the sources of PAHs [21, 22, 23]. It is commonly noted that a ratio Phe/Ant>10 indicates petrogenic PAHs, whereas low Phe/Ant ratio (<10) implies combustion sources of pyrolytic PAHs. Anthracene was only detected in D1, where a Phe/Ant ratio of 2.19 was measured, indicating a pyrolytic source, as expected according to the history of burning at many dump sites in Lebanon [2]. In general, Flt/Pyr ratios >1 originate from pyrolytic sources while ratios of <1 infer petrogenic source [24]. Both ratios were found in different water samples indicating the presence of mixed pyrolytic and petrogenic sources.

Furthermore, it is has been widely reported that a Ant/(Ant + Phe) ratio <0.1 indicates a petroleum source, whereas a ratio >0.1 implies an extensive combustion of wastes [20, 25]. Our investigations revealed that the ratio was 0.313 at D1 site, signifying that the source of PAHs is extensive combustion of wastes. Furthermore, a ratio of Flt/(Flt + Pyr) < 0.4 signifies a petroleum source; however, a ratio >0.5 infers straw and coal combustion. An intermediate ratio between 0.4 and 0.5 indicates liquid fossil fuel combustion [23]. Our findings showed that Flt/(Flt + Pyr) ratios ranged between 0.438 and 0.619 suggesting that PAHs contamination of water samples collected from Deir Kanoun dump and canal were generally derived from combustion activities. Hence, it is suspected that the open burning of waste might be the main source of PAHs.

During summer season, similar patterns of organic compound composition in water samples were observed. DEHP and bisphenol A were detected only in water samples collected from C1 site and their levels were 72.3 and 0.154 μg/L respectively. Moreover, total PAHs ranged from 286.55 ng/L (at C1) and 922.68 ng/L (at C2), where most of these PAHs were found to be of LMW. Carcinogenic PAHs represented 0.42–1.5% of total PAHs. Flt/Pyr ratios were >1 in samples from C1 and C2 sites indicating pyrolytic sources, and Flt/Flt + Pyr ratios were >0.5 implying combustion activities as sources of such contaminants.

Neither anthracene, acenaphthlene, acenaphthylene, benzo(a)pyrene, nor benzo(g,h,i) perylene was detected in samples collected during summer season. Also, neither DTT, PVC nor Di-n-butylPhthalate (DBH) was detected in all samples collected during this study (data not shown).

## 4 Discussion

Lebanon is a Mediterranean country that has been suffering from a continuous waste crisis over the two past decades [6]. Lately, the influx of Syrian refugees to Lebanon had led to increased water demands (70 million cubic meters) and solid waste production (16% from refugees) [4]. These increases in waste production, along with the improper waste management from the Lebanese authorities, resulted in more unresolved problems of environmental pollution [7, 8, 9].

Deir Kanoun Ras El Ain and Klayleh are southern Lebanese villages that harbor ∼1750 Syrian refugees (11.5 % of the total population in each village) [5]. Deir Kanoun Ras El Ain possesses significant underground water resources that are used for both domestic and irrigation purposes (1255 ha). It also hosts one of the worst dumps in the country, where medical, industrial and household wastes are deposited, and where the dump leachates flow into a canal that runs alongside [10].

The high concentrations of heavy metals and organic compounds reported herein may exert dangerous effects on both the ecosystem and human health [2]. Through oral administration of contaminated water, heavy metals can be transported to humans, accumulate and exert significant toxicity [5, 26]. Therefore, serious health and environmental issues regarding heavy metal contamination are of great concern, especially in developing countries where several forms of pollution are widespread.

Pb, Cd, As, and Hg are serious metal pollutants that, when present in high levels, lead to metabolic disorders in most living systems [26]. Arsenic, lead and mercury come in the first, second, and third positions in the Comprehensive Environmental Response, Compensation and Liability Act (CERCLA) list that enlists the 275 most dangerous substances in 2011 [27]. Cadmium comes in the seventh position in this list. Our findings indicate that Pb, Cd, Hg, and As were the most serious metal pollutants in water samples collected from Deir Kanoun dump and canal. Similar patterns of heavy metal concentrations were observed during winter and summer seasons except for Pb, whose concentration in water samples was one order of magnitude higher in summer than winter. This may be the result of slower currents of water in summer that allow the settlement and accumulation of this metal in water. More importantly, the high levels of heavy metals in all sites are cause for concern as they highly exceeded the FAO and CCME permissible limits, except for Pb. These findings are of particular concern because the canal's water is used in various forms by inhabitants and refugees living near the dump. The elevated levels of metals in the canal water further implicate the extent of metal input from dump leachates as well as from anthropogenic activities that contributed to high metals at C2 site, which is far from the dump.

These high levels of heavy metals found in water samples are attributed to the types of wastes dumped in Deir Kanoun Ras El Ein landfill. Lead sources may include batteries, pesticides, plastics, paints, and ceramic glaze, and its accumulation in the body leads to anemia, neural and renal damage, delay in growth and learning disabilities [28]. The major sources of As include pesticides, wood preservatives and glass products, whereas the major sources of Cd and Hg are industrial wastes, pigments/paints, fertilizers and pesticides, electronic wastes, fossil fuels, car battery wash wastewaters and plastic wastes [29, 30]. In humans, even low levels of Cd, As and Hg are considered toxic as they may cause many deleterious effects, including liver and kidney damage, bone and cardiovascular diseases, stomach and intestinal irritation as well as cancer [31].

Our findings are similar to a previous study conducted by Olafisoye *et al.* that showed high concentrations of heavy metals in water and soil samples around an e-waste dump in Nigeria during wet and dry seasons. Those high concentrations of heavy metals exceeded maximum permissible levels [32]. Likewise, a study by Tiwari *et al.* showed high concentrations of heavy metals present in water leachate samples collected from an industrial region of central India that is characterized by dumping and disposal of industrial solid wastes [33].

The analysis of phenols, phthalates and PAHs reported in this study provides additional insight into the identification of wastes' sources. The PAHs wastes can come from either incomplete combustion or petroleum sources, whereas the sources of phenols and phthalates involve consumer products and building materials [34]. These compounds have been shown to exert acute toxic and carcinogenic effects in living systems. They may also lead to pulmonary, respiratory, genetic, hepatic, gastrointestinal, reproductive, neurotoxic, and developmental problems [35, 36, 37, 38]. In addition, their presence in water leachates may pose a serious threat to the receiving water quality and subsequently to the health of individuals using these resources for drinking and irrigation purposes [39, 40]. The composition of organic compounds in wastewaters, including dump leachates, may vary in composition depending on the type of waste, year of deposition and the age of the landfill [41]. Our results revealed the presence of phenols, phthalates and PAHs in water samples collected from different dump and canal sites. In fact, the relatively low levels of organic compounds are due to seasonal variations of canal flow, which caused the dilution of pollutants.

In summary, results of assessing water samples from different dump and canal sites indicated that petrogenic and pyrogenic wastes contributed to the heavy metals, phthalates, bisphenol, and PAHs pollution to the studied environment. Contaminants exposure may induce adverse impacts on the health of inhabitants living near to the dump. Because of the high heavy metal concentrations, water from the canal should not be used for agricultural purposes. In January of 2017, the Lebanese cabinet prepared a summary policy on Integrated Solid Waste Management [42], and the environment minister formed a committee on waste management [43], which includes a civil society representative. Thus, it is recommended that the Lebanese authorities regularly monitor the levels of heavy metals in water and take more efficient and effective actions to deal with the current waste management issues.

## 5 Conclusion

Due to the lax environmental policies in Lebanon, environmental problems generated from uncontrolled dumping activities became more complex and serious especially in Deir Kanoun Ras El Ein and the surrounding villages. The present study revealed that water samples collected from dump and canal were heavily polluted by heavy metals, DEHP, bisphenol A, and PAHs caused by pyrogenic and petrogenic sources. The study indicates that the concentration of the found heavy metals and organic compounds were all far above the maximum tolerable levels set by different guidelines, except for Pb. The findings might contribute to a greater awareness of the serious implications and health hazards of dumping wastes in this landfill that is accompanied by a polluted canal used to irrigate agricultural lands. In conclusion, it is highly recommended to propose more efficient and effective waste management policies to resolve this serious environmental problem.

## Declarations

### Author contribution statement

J. Borjac-Natour: Conceived and designed the experiments; Analyzed and interpreted the data; Wrote the paper.

M. El Joumaa, D. Blake: Wrote the paper.

R. Kawaach, L. Youssef: Conceived and designed the experiments; Performed the experiments; Analyzed and interpreted the data.

### Funding statement

This work was supported by the U.S National Academy of Science PEER cycle 5 program (grant number 5-56 and Grant Award Number AID-OAA-A-11- 00012).

### Competing interest statement

The authors declare no conflict of interest.

### Additional information

No additional information is available for this paper.
